# The Hospitals Association

**Published:** 1897-11-06

**Authors:** 


### THE HOSPITALS ASSOCIATION.

### THE DISTRIBUTION OF THE HOSPITAL SUNDAY AND SATURDAY FUNDS.

A very largely-attended and most representative meeting was held under the auspices of The Hospitals Association at the board-room of the Charing Cross Hospital on Thursday, the 28th ult., to hear from Sir Sydney H. Waterlow and Mr. R. B. D. Acland the methods of distribution adopted by the Metropolitan Hospital Sunday and Saturday Funds respectively. The President of The Hospitals Association, Mr. H. Cosmo O. Bonsor, M.P., took the chair, and those present included the Earl of Stamford, Sir Henry Burdett, Sir Owen Roberts, the Revs. Canon Graham, Prebendary Kitto, Professor Wiltshire, J. G. Grundy, M. S. A. Walrond, D. Anderson, and Brooke Herford, Drs. T. D. Acland and E. Hayward, Lieut.-Colonel Lambert Mears, Lieut.-Colonel E. Montefiore, Surgeon-Colonel Howard, Surgeon-Lieut.-Colonel J. Ince, Colonel Hamond, Captain J. Cundy, Messrs. T. Bryant, A. Cohen, N. Cohen, A. G. Sandeman, J. Astley Bloxam, F. Willcocks, Hamilton Hoare, J. Rolls Hoare, A. Willett, and A. F. Durham, the Rev. Nathaniel Bromley (King's College Hospital), Messrs. Henry Williams (Guy's), T. Ryan (St. Mary's), C. W. Thies (Royal Free), S. Quennell (Westminster), H. N. Custance (Hospital Sunday Fund), W. G. Bunn (Hospital Saturday Fund), G. A. Cross (London Homœopathic), G. A. Scudamore (Samaritan Free), A. Watts (Queen Charlotte's), B. Burford Rawlings (National Hospital for the Paralysed, &c.), and Arthur Hayes (Metropolitan Convalescent Institution), Miss Baxter (New Hospital for Women), and Miss Marion Ritchie (Clapham Maternity).

The Chairman, in opening the proceedings, said that Sir Sydney Waterlow needed no introduction at a meeting of that description. He was Lord Mayor of London when the Metropolitan Hospital Sunday Fund was formed twenty-five years ago, and ever since then he had acted as chairman of the Distribution Committee of that Fund.

### The Distribution System of the Sunday Fund.

Sir Sydney H. Waterlow, Bart., read the following paper: Several of the friends and warmest supporters of the Hospital Sunday Fund have asked me to attend here to-day and explain to them, and through them to the public generally, the system upon which the money collected on Hospital Sunday is divided between the hospitals, dispensaries, convalescent homes, and other institutions applying for an award. Collections for the benefit of the Fund have now been made for 25 years successively, and the total sum collected to July 30th, 1897, was £913,482. It is not surprising that the friends of the movement and the public generally should be desirous of understanding the manner in which this large sum has been divided among the numerous institutions applying for a share of the Funds. This desire has been no doubt further stimulated by the fact that probably half a million of money will be raised this year by the Prince of Wales's Hospital Fund for London. When in February last His Royal Highness made his appeal for contributions to this Fund he stated: "I am aware that the task of distributing the funds when collected will not be without its difficulties. It may be desirable in the future to seek the assistance of representatives chosen by the hospitals, but for the first year we shall rely upon the co-operation of the Distribution Committee of the Hospital Sunday Fund."

### The Composition of the Distribution Committee.

The Distribution Committee consists of the Lord Mayor, as president, and ten members chosen annually by the Council; some of the members have served upon the committee for more than 20 years. The Fund was started at the Mansion House in the year 1873. I was the first president and treasurer, and have been chairman of the Distribution Committee from the commencement. I have rarely missed attendance at any of the meetings, and consequently am perhaps more responsible than any other person for the preparation of the laws by which the distribution of the money is governed, and for the interpretation which has from time to time been placed on them. All the members of the committee have necessarily gained considerable experience in dealing with the important duties cast upon them. We have upon the committee a very eminent surgeon, and a physician of high standing in the City of London; the professional knowledge of these two gentlemen has been of great assistance to the committee. It has also been thought right not to elect upon the committee any gentleman who held office in connection with any one of the hospitals applying for an award. For some years after the collections were commenced, neither of the three endowed hospitals—St. Bartholomews, St. Thomas's, or Guy's—applied for, or received, any award. The first application from Guy's was in the year 1888. No award has hitherto been made to St. Thomas's, as the governors of that hospital did not see their way to deliver copies of their accounts made out in the form prescribed by the hospital authorities and the Council, and certified by a public accountant. It is, however, understood that the treasurer of St. Thomas's will have the accounts of his hospital prepared in the prescribed form, and that an application will be made next year for a share of the Hospital Sunday Fund. St. Bartholomew's Hospital has at present no need for any assistance from the Fund.

### The Laws Governing Their Work.

It should be borne in mind that the work of the Distribution Committee is governed and controlled by a series of laws passed by the constituents of the Fund, who meet annually at the Mansion House as representatives of the congregations contributing the money on Hospital Sunday. These laws direct that only those hospitals and dispensaries which are managed by committees duly constituted, shall be allowed to participate in the Fund. The awards to hospitals, &c., shall be primarily based on the average total expenditure of each institution for the last three years, after deducting therefrom: (1) A sum equal to the income derived from endowments and realised property; (2) the amount received in legacies exceeding £100 each, unless such legacies have been necessarily spent to meet the current expenditure of the institution; and (3) the expenses of management. In every case the merits and pecuniary needs of the institution concerned shall be fully inquired into and considered by the Distribution Committee, and the award made shall be determined in accordance with the judgment of that committee upon such merits and needs, provided that in no case shall the grant be reduced below the arithmetical basis, or withheld, until a conference shall have been sought with the managing committee of the hospital affected. The payments made by or on behalf of patients shall be dealt with, in each case, as the committee of distribution may see fit. This was found necessary owing to the very great variation in the amounts and in the circumstances under which these payments are made. No institution, to the benefits of which admission can only be gained by election from the general body of subscribers, shall be eligible for grants from the Fund. The committee shall have power to set aside 5 per cent. of the total amount of collections for the purpose of surgical appliances. There are several other minor provisions, but they have no material effect on the amount awarded. A careful consideration of these laws clearly indicates that the Distribution Committee are to recommend awards to each hospital, &c., in accordance with its needs and merits, subject to the definite directions for arriving at these important factors.

Every institution desirous of applying for an award has to send to the secretary of the Fund, not later than February or March in each year, an application to participate. This must be accompanied by a correct copy of the printed report, and of the receipts and expenditure of the institution, made out upon the prescribed form agreed upon between the Council of the Fund and the managers of the leading London hospitals. These accounts are then required to be analysed on a form supplied for the purpose in duplicate. As these forms are all alike, the comparison of the relative pecuniary needs is not difficult. The expenditure is divided under two heads—maintenance and management. Maintenance includes provisions, surgery, domestic expenses, establishment charges, rates and taxes, rent (if any), salaries and wages (except for those persons engaged in the administration), miscellaneous expenses, with a separate item for extraordinary repairs and new buildings, which is always separately considered. The administration or management includes official salaries and commissions, official printing, postage, telegrams, &c., official advertisements, law charges, incidental management expenses, with a separate item for appeals and festival expenses, separately dealt with. It will be seen that maintenance, in other words, includes all the money spent in the care and cure of patients; while the administration is the cost of the office management controlling the work. The festival expenses I will hereafter refer to. The income is divided under two items—charitable revenue and proprietary revenue. The charitable revenue includes subscriptions, donations, the Sunday and Saturday Funds, church collections (if any), festivals, legacies under £100, or exceeding that sum, but absorbed in current expenditure. The proprietary revenue, or secured income, includes interest on legacies exceeding £100, when not required for current expenditure, dividends, interests, rents, &c., from invested property, fees paid by nurses, and patients' payments.

### How the Needs and Merits of a Hospital are Assessed.

In order to arrive approximately at the needs of the hospital, the average of the moneys spent in maintenance and management in each of the three past years is carefully calculated. From the total expenditure in each year we then deduct the proprietary, or secured annual revenue. The money expended on the average of three years in the treatment and support of the patients, less the proprietary revenue and the expenses of management is regarded as the financial needs of the hospital, which have to be provided from year to year from the public and from uncertain sources. This is called the basis of the award, and is raised or lowered at the discretion of the committee, as soon as they have determined the merits of the particular institution. The task of arriving at the merits is no doubt more difficult than determining the financial needs, and rests to a greater extent on the discretion of the committee. Detailed information, including the number of beds, the average number occupied daily during the last year, the average number of days each in-patient was resident, the average cost of each in-patient per week, and the average cost of each out-patient, as far as this can be calculated, is given for the past year; the average number of in, out, and lying-in patients, both counting renewals, and without renewals; and also the total number of attendances for the past three years is all laid before the committee. Although the figures thus furnished in no way enter into the arithmetical calculation of the needs of the hospital, they have to be most carefully considered when determining the merits: the average cost of each in and out-patient, and the numbers of beds occupied, as compared with those unoccupied, varies so much in different hospitals. Let me take eight of the large general hospitals, viz., Charing Cross, Guy's, King's College, London, St. Mary's, Middlesex, University, and Westminster, the management in which may be regarded as satisfactory. The cost of each in-patient per week varies from 26s. to 36s., while in smaller hospitals and special hospitals it reaches as much as £2 8s. In determining whether the amount is or is not excessive, the committee have naturally to bear in mind the nature of the work carried on, the character of the diseases dealt with, especially in special hospitals, as some classes of patients, if efficiently treated, cost much more than others. The percentage which the money spent on administration bears to the money spent on maintenance is necessarily a very important factor in determining the relative merits of different hospitals. Taking the eight hospitals previously referred to, the percentage of management to maintenance varies from 3·883 per cent. to 13 per cent. This may be to some extent accounted for by slight variations in the manner in which the accounts are stated, but not materially. In the special and smaller hospitals the percentage varies from 2 per cent. to 31 per cent. It must, however, be borne in mind that in smaller hospitals the expenses of management are unavoidably much larger than in the larger hospitals; but there can be no doubt that money contributed to the large well-conducted hospitals, goes very much farther, and secures the efficient treatment of a larger number of patients than in the smaller hospitals. The committee have, however, always considered that when comparatively small hospitals are erected in outlying districts at some distance from existing hospitals, they ought, if well managed, to have every encouragement.

A lithographed abstract analysis of the accounts of each hospital is given to every member of the committee, attention is called to the particulars I have referred to—no decision is recorded until these points have all been discussed, a vote is then taken in each case.

It should always be borne in mind that whenever the committee are of opinion that the information before them does not seem to justify them in recommending an award equal to the natural basis disclosed by the figures, they are required by Rule 5 of the Laws of the Constitution to ask for a conference with the managers of the hospital affected. During the present year conferences were invited with the managers of 38 institutions, and in 30 cases deputations were interviewed. In several cases the basis was materially reduced, the managers promising that the defects complained of should be remedied in the future, and in two cases the management was so bad that no award was recommended.

The item—cost of appeals, and festival expenses—is one which gives the committee much trouble, and it cannot be dealt with upon any hard and fast line. Speaking however, for myself, I have always held that where these expenses result in bringing into the treasury of the hospital only 50 or 60 per cent. of the sum contributed by the persons present at the festivals, they ought not to be encouraged.

I have endeavoured to make the method adopted by the Distribution Committee as clear as possible. I will now give two illustrations, or examples, with the view of rendering my statement more intelligible.

London Hospital.—The average total expenditure for 1894, 1895, and 1896, after deducting each year the proprietary revenue, was £44,884. From this sum we deduct the average cost of management for the same years, viz., £2,277, leaving £42,607 as the needs of the hospital to be collected annually from the outside. This forms the primary basis, to be raised or lowered on the merits, at the discretion of the committee.

St. George's Hospital.—The average total expenditure for 1894, 1895, and 1896, after deducting each year the proprietary revenue, was £18,441. From this sum we deduct the average cost of management, £1,481, leaving £16,960 as the needs of the hospital to be collected annually from the outside. This forms the primary basis, to be raised or lowered at the discretion of the committee.

The question as to the continually increasing number of out-patients at our large hospitals has been much discussed of late, and these discussions have disclosed a great diversity of opinion, but as I have previously stated, the number of out-patients treated at each hospital has hitherto been regarded by the Distribution Committee simply as evidence of the amount of work done in treating the sick poor, and has not been an important factor in determining the amount of the awards to each hospital. I have, therefore, refrained from dealing with it here.

### Summary of the System.

The system we are discussing may be very shortly summarised thus: By means of the analyses of the accounts to which I have referred, the committee are enabled to arrive at the sum of money spent by each hospital on the average of the past three years in the treatment and support of the patients. Deduct from this the proprietary revenue, or ensured income, and you have the figures representing the needs of each hospital. These figures form the primary bases of the awards. The merits are then discussed on reference to the accounts, and from information placed at the disposal of the committee from various sources. If there is no fault to find with the management, the figures forming the bases are confirmed or raised; if the management is unsatisfactory a conference is invited with the managers. The money available for distribution is then divided in accordance with the figures of the bases. It has varied during the last 25 years from 1s. 10d. to 2s. 2d. on each basis, or approximately on each pound needed for the support of all the hospitals, &c. The variation is governed by the amount collected each year. It should also be remembered that the money is distributed within a few weeks after it has been collected. I have always felt that those who give the money intend it for the relief of the sick poor in the year in which it is contributed.

I think I am justified in saying that this system of distribution has been thoroughly successful and satisfactory to the public. The awards have for the last 25 years been submitted for the approval of the Council, consisting of 100 members, at a meeting in the Mansion House, and published in the London Press. On one occasion only has any alteration in the awards recommended by the committee ever been thought necessary. If we take the average of the last 25 years of the contribution to the hospitals from the Sunday Fund at 2s. in the pound of the needs of each institution applying, it will be seen that the Fund provides only one-tenth of the cost of the treatment and support of the sick, leaving the other nine-tenths to be provided by subscriptions, donations, &c., from the public, and from legacies or other sources.

But this is not by any means the only benefit which the hospitals and the public have derived by the action of the Committee of Distribution. With the assistance and full concurrence of the secretaries and officials of the hospitals all the accounts of income and expenditure are now prepared on one uniform plan, and certified by a public accountant, before they are subjected to the detailed criticism of the committee. This arrangement has undoubtedly in many cases ensured much greater economy and an improved management of the Hospital Funds. Again, my experience of the last 25 years has much strengthened my early conviction that an annual sermon in the churches and chapels, followed by a collection, was the best, and likely to be the most permanent method of raising money for hospitals, for it is well that on one Sunday in each year all the ministers and the whole population in the churches and chapels should forget for the moment denominational differences, and join hearts in one Christian duty, upon which all are agreed, viz., the duty of relieving the sick poor, each one as far as he is able; and I feel sure that a large number of those who contribute small sums at the collection on Hospital Sunday are so impressed with the sermons that they give large donations at a later period or leave legacies for hospital purposes. (Applause.)

In conclusion, I wish it to be distinctly understood that I alone am responsible for the opinions expressed in this paper. It has never been read to my colleagues on the committee, and therefore I have no authority to speak for anyone but myself.

### THE DISTRIBUTION SYSTEM OF THE SATURDAY FUND.

Mr. R. B. D. Acland (chairman of the Hospital Saturday Fund) opened the discussion. He said: It affords me the greatest possible pleasure to address you on the system of distribution adopted by the Hospital Saturday Fund, for these reasons, first, that I am one of those who believe that it is only by criticism, based upon full knowledge of the facts, that any advance can be made in work of this sort; and, secondly, because I believe that a meeting of this kind can but have one result—a result which has been very near to me, a result which I have been very anxious to attain for very many years—and that is that the system of distribution of both the Hospital Saturday and Sunday Funds, and indeed the Prince of Wales's Fund, should be the same. (Hear, hear.) That has long been an ideal of mine, and I have taken whatever steps I have thought to be possible to attain to it, and I look upon this meeting as the greatest step taken in that direction since the two Funds were inaugurated.

### Two Difficulties.

Before going into the details of our system of distribution, I wish to call attention to two difficulties, the first one which must confront any distribution committee, whether it be that of the Sunday Fund or that of the Saturday Fund, and the second a difficulty peculiar to the Saturday Fund itself. The first is the extreme difficulty of getting at the actual numbers of the patients who attend the hospitals. That is a bold statement to make in a hospital board-room, but anyone who has to serve on a distribution committee will bear out my statement. The second difficulty is, as I have said, peculiar to the Hospital Saturday Fund. Most present know that we are a working man's organisation, and the Saturday Fund has for the last 23 years been trying to work out its own salvation in the way of collecting and distributing funds for the hospitals. It has received no assistance whatever—I am not complaining, I am only stating a fact—from people with a peculiar knowledge of hospital work, and if there are defects in the system—and I have no doubt that there are, for I do not imagine that it is a perfect system—it is due to the fact that we have had to work it out from paper rather than from an actual knowledge of the necessities of hospitals. This has had another effect on our system of distribution, and that is this: It has been necessary for us as we have gained experience to codify the rules which have suggested themselves to us, and you will find when I go on to describe the system to you that it is far more detailed and far more red-tapey, if I may use the term, than the system of the Sunday Fund. The Sunday Fund system may be summed up in this way—Given your arithmetical basis as that to which a hospital may attain, it is left to the distribution committee in its own unfettered discretion to say whether a grant shall be made on that basis or not. That may be a very excellent plan where, as in the case of the Sunday Fund, the Distribution Committee is composed of men with the fullest knowlege of hospital administration. It would, however, be a very bad system indeed if it was applied to the Hospital Saturday Fund, where the Distribution Committee is composed of men who have not that knowledge which is essential to make use of the enormous power which is thus placed in the hands of the Distribution Committee.

### Institutions Receiving Grants.

I will now proceed to deal in detail with the system which we have adopted, only saying this, that we thoroughly overhauled it this year, and it is now a great deal more perfect than it was a year or two ago. We had first of all to decide what were the institutions to which grants should be made. We have found from time to time that institutions have applied for grants which are neither hospitals, dispensaries, nor really medical charities, and, therefore, we have thought it necessary to define the institutions that are entitled to a grant as those which are solely medical charities and those that treat without election the sick poor. They include all hospitals properly so-called, dispensaries, convalescent homes, and nursing institutions, which, I understand, the Sunday Fund takes no account of at all. We have done that in order to exclude bodies like medical missions and the Salvation Army, and bodies of that kind, which we found were making applications to us on the ground that they were doing work amongst the sick poor. Very estimable institutions, I have no doubt, but not institutions to be supported from a hospital fund.

### The Amount to be Distributed.

The next thing we have to do is to arrive at the amount to be distributed. We first, therefore, deduct a certain percentage for what I may call the outside work of the Hospital Saturday Fund. At the present day the Saturday Fund is not a mere collecting and distributing body. We are doing a great deal of work entirely outside that description. We are doing a great deal of ambulance work in the way of instructing the men in the workshops in "first aid," and in supplying them with the necessaries for rendering that "first aid." We are doing very considerable work in supplying necessary surgical appliances and instruments where accidents have occurred, and also in enabling people who cannot afford to pay the whole of the money necessary to take them to a convalescent home, but who, at the same time, do not wish to be objects of charity, to go down at their own cost, we advancing the money necessary in the first instance and allowing them to pay us back by instalments. Therefore we deduct, before we arrive at the amount to be distributed among the hospitals, six per cent. for surgical appliance work, eight per cent. for the purchase of letters, a sum not exceeding one per cent. for Morley House Convalescent Home (which was practically founded by the Hospital Saturday Fund, and which we desire in some way to give additional support to as long as that support is required), and other deductions for special cases, which altogether makes a deduction of from 13 to 14 per cent. of the total sum collected. The balance is divided among the different hospitals, dispensaries, convalescent homes and nursing institutions, in accordance with the rules which I will now explain.

### The Method of Distribution.

The basis of our whole scheme, with the exception of one particular, very much resembles that of the Hospital Sunday Fund. It is based on the relief afforded, the economy practised, and the efficiency shown. I am going to say nothing about the relief for a moment, as that is a point on which above all others we differ from the Hospital Sunday Fund.

### Method of Assessing Efficiency.

We attempt to arrive at the efficiency of a hospital, as far as we can discover it, by a series of questions asked with a view to showing the probability of a hospital being properly managed. The way in which we do that is this. We ask a series of questions with which any hospital secretaries who are present will be painfully familiar, but which are all asked with a serious object. The questions are of this sort: "Have you a committee of management?" "How is it elected?" "Is there an independent finance committee?" "If so, by whom is it appointed?" "Is there an annual meeting of the governors and subscribers?" If so, we want to know the date, in order that our representative may go. We also ask how it is called and whether reporters are admitted, so that we may know that it is a public meeting. There are various other questions, which I will deal with in a moment. The answers to questions of the kind that I have read out to you will give outsiders a certain knowledge of the probability of a hospital being well managed. What it all comes to is: Is there efficient control, and if so, are the people who control properly elected, and what guarantees are there that the control is effectually exercised? There are certain other questions which represent more or less distinctly the Hospital Saturday view of the matter. Thus: "Are you willing to give to the Hospital Saturday Fund, in return for grants, letters in the same proportion as you give to the ordinary subscribers?" "May patients be visited by friends on Sundays, and, if so, at what hours?" There used to be a question as to whether or not evening attendances were allowed, but that has now disappeared. Those questions represent the view of the working man. I am not here to attack or to defend it. At the same time, it appears to me to be a reasonable view from the point of view of the working man who subscribes the money. He thinks that a guinea contributed by himself and his shopmates is worth just as much to an institution and is just as likely to do as much good to the sick poor as a guinea contributed by the richest man in England, and if that is true the average working man thinks that he is entitled to the same advantages, if they be advantages, to which the very rich man who out of his richness gives a guinea, is entitled. That is a view which I think it is right I should put before you, and it is one which I think is reasonable enough. The use which is made of these questions is this. A maximum number of marks—15—is given for efficiency. Each question which is answered adversely or wrongly, which shows that the hospital is not likely to be well managed, will cause a hospital to lose a mark, and then, when the total number of marks are awarded, a hospital works out at so many marks for efficiency. I do not trouble you, as the time is short, with the distinction we make between the different sizes of hospitals, but we try to secure that a hospital which is small and should only get a small grant shall not, by the economy and efficiency awards, get too large a proportion of the fund which is at our disposal as compared with the larger hospitals.

### Method of Assessing Economy.

I now come to economy. The Hospital Sunday Fund practically leaves this in its entirety to the distribution committee. For reasons which I have explained, we are not able to do that, so we have tried to formulate a series of rules which will have the effect of fairly working out the economy shown by the different hospitals. Our system is shortly this: It is all based upon the cost per bed in an institution and the cost per out-patient as shown by the different returns made by the hospitals. Now it is quite clear to anyone who knows anything about hospital work that the cost per bed in a hospital, or the cost per out-patient, must vary according to the kind of work which is being done in that hospital. It would not be fair to compare a general hospital with a hospital say like the Brompton Hospital for Consumption, or perhaps the Cancer Hospital, and so what we have tried to do is to group together the hospitals in classes, and then we have worked out averages for the cost per bed and the cost per out-patient for each one of these classes. The classes are five in number, if we exclude convalescent homes, cottage hospitals, and dispensaries, and these averages have been worked out after a careful comparison extending over a number of years as to the different costs of these items as shown by the returns to us. The classes are, first, general and ophthalmic hospitals; the second includes hospitals for women and for stone, hospitals for diseases of the throat, cancer and chest hospitals, and hospitals for paralysis, fistula, and diseases of the skin; the next contains children's and dental hospitals; the fourth includes lock and orthopædic hospitals; and the fifth lying-in hospitals. The cost per bed is again divided into four classes in this way. Take the general hospitals, for instance. If the cost per bed is less than £60 no mark is lost, if it is £60 and under £70 one mark is taken off, if it is £70 and under £80 two marks are taken off, if it is £80 and under £90 three marks, and if it is £90 or over, four marks. Again we take a comparison between the cost of management and the cost of maintenance, and here again it has to be worked out arithmetically. We take the relation between management and maintenance, as it is taken by the Hospital Sunday Fund, and then instead of leaving it to the discretion of our Distribution Committee, we say that where the management costs 10 per cent. of the maintenance the hospital loses one mark, where it is 13 per cent. it loses two marks, where it is 16 it loses three, 19 four, 22 five, and where it is over 22 it loses six marks. So it is quite possible under this system that a hospital which is extremely expensively managed would get no marks at all for economy.

I ought to have explained how we divide the money which we have got to distribute so as to bring in these considerations of relief, economy, and efficiency. We do it in this way. Of the amount available for distribution five-sixths is to be awarded for relief and efficiency, and one-sixth for economy. Then the marks which are given under these different heads are divided into the money which is to be distributed, and so you get exactly the value of an economy mark, a relief mark, or an efficiency mark.

### Method of Assessing Relief.

Now I turn to the relief part of our system of distribution, and here I may say that I really think that the difference between the Saturday and Sunday Funds is smaller than it appears at first sight to be. By deducting the proprietary revenue from the expenditure, as is done by the Sunday Fund, some account must be taken of the work which is done. The Saturday Fund does this too, but it does it more directly. We count heads; we see the number who are actually treated, and we allow a certain number of these to count towards marks. I say to count towards marks because it is obviously necessary in working out a system based as ours is on the number of patients treated that some consideration should be given to the income which is in the possession of the hospitals, otherwise St. Bartholomew's, according to a system of merely counting patients, would get a far larger grant than perhaps any other hospital in London, whereas, as a matter of fact, it does not require a grant at all. The system we have adopted is this. We only count as patients for the purpose of relief-marks those who are not paid for either by patients themselves or by the proprietary revenue possessed by a hospital, and that immediately, of course, cuts out a hospital like St. Bartholomew's, which requires no income from charitable sources at all. We go further; we say there are a certain number of hospitals in London which are really paying institutions—I will not mention names—and so we have made a rule that where a hospital receives 75 per cent., or upwards, of the cost of maintenance from its patients they must not receive an award at all. We therefore put them in the category of paying hospitals, and if any of our subscribers want to go to that hospital we buy the letters and put the matter frankly on a commercial basis. Then we come to the out-patient department. One more word, though, I have to say first of all as to the relief-marks for in-patients. We have been seeking about for some time to find out how it would be possible for us to help hospitals like St. Thomas's, which have been obliged to close some of their wards for fear of running into debt. It is not easy to devise a system which shall be fair to other hospitals, but after considerable thought we have come to the conclusion that we may do it in this way, by asking for a return of the daily average number of beds occupied and the number of beds in the hospital which could be occupied if the hospital were in a position to fill them. We give, in the case of general hospitals, one mark for every two beds which are not occupied for the reason which I have stated. Therefore, we give to those hospitals which are so poor that they cannot make full use of their buildings, and which are so careful that they will not run into debt, a bit of a pull over the hospitals which are kept full by running into debt, or by having a sufficient sum of money from their subscribers to keep all their beds going. Now I come to the out-patient department. We award marks for the number of patients who are treated, not for the number of attendances. We give, roughly speaking, one mark for every 2,000 to 800 patients. I say for from 2,000 to 800 for this reason. It is clear that at some hospitals—take, for instance, a consumption hospital—the average number of attendances by an out-patient will be much greater than it will be at a general hospital, especially if you take into account the number of casualty cases treated at a general hospital; and so here again we have tried to divide the hospitals into classes which represent more or less accurately the amount of work which is done in relation to each out-patient. Where, for instance, it is shown in the returns that each out-patient attends on an average twice, that hospital would get a less proportion, a less payment for each out-patient than a hospital where it is shown that each out-patient attends five times. It is an attempt to deal with the matter from the point of view of the work which is done by each separate hospital.

### THE DISCUSSION ON THE TWO PAPERS.

As no one seemed inclined to carry on the discussion,

Sir Henry Burdett said that, although he had not intended to speak, he thought it would be but a poor compliment to both Sir Sydney Waterlow and to Mr. Acland if no discussion took place. The best way of showing their gratitude to those gentlemen for the great trouble they had taken would be by taking up any points in the two systems which they wished to have further light thrown on. In order to initiate discussion, he would like to ask Mr. Acland two questions. First, was he right in saying that under the Saturday Fund's system only six marks were taken off when the management expenses amounted to over 23 per cent. of the amount spent on maintenance? By that rule if a charity spent 11¾d. out of every shilling on management expenses they would apparently only lose six marks. Secondly, Mr. Acland had told them that before a single penny was given to the hospitals 8 per cent. was taken off for the purchase of letters. If any progress had been made anywhere in hospital affairs it had been made in the matter of abolishing hospital letters, and he (the speaker) could not see any justification for any fund which raised money for distribution amongst charities setting apart a portion of this money for the purchase of letters, when it was known that the cost of a patient in a hospital was greatly in excess of the amount charged for a letter. It was conceivable that the Hospital Saturday Fund's system on this point might in practice so work out that not only was no money given towards the support of a hospital, but the Fund might possibly be a charge on the offerings of the public. In raising these two points he wished it to be clearly understood that he was not criticising the Saturday Fund, for all owed Mr. Acland much for his able and devoted labours as chairman of this Fund.

The Earl of Stamford asked whether in the systems just explained the number of out-patients counted in the allocation of grants, and if so, to what extent? He also wished to ask Mr. Acland, if the working man looked upon his and his mates' subscription of a guinea to a hospital as giving them a claim individually to free hospital treatment, where was the moral iniquity in a rich man, who contributed, say, £50, going to the hospital also and expecting to be treated free? That, in his opinion, cut at the root of the abuse of the hospitals, and he feared that if the notion got about that hospital subscription was not charity but insurance, the whole basis of the voluntary system would be undermined.

Rev. Prebendary Kitto said that the initial difference between the two funds was that the Saturday Fund was a system of insurance, and the Sunday Fund a system of charity. For that reason amalgamation was impossible, although it was possible that both funds might in the future adopt a similar system of distribution.

Mr. Thomas Ryan (St. Mary's Hospital) said that one point he would like to make was this. The Saturday Fund's system gave the full number of marks for economy to a hospital at which the cost per occupied bed was £60, and deducted marks where it was £70, £80, or more. It did not in the slightest degree follow that an institution which spent £60 per bed was more economically managed than one which cost £70. It was administered more cheaply, but not necessarily more economically. Mr. Acland had also said that the working man's guinea was worth as much to the hospitals as the rich man's guinea. That was not so, however, because the basis on which letters were issued by the hospitals was that the rich man would probably not use his letters, while in the case of the working man the letters were obtained with the full intention of using them. As to the Sunday Fund, he thought the method of arriving at the merits of an institution appeared on the face of it to be slightly what he might call slap dash.

Mr. Conrad W. Thies (Royal Free Hospital) suggested that the two funds might well submit their systems to a small committee of hospital secretaries, which might be useful in giving practical advice on the many difficult points which required further consideration. It struck him as singular that neither of the two funds had any committee of visitors, whose duty it would be to inspect the hospitals.

Rev. Nathaniel Bromley (King's College Hospital) could not understand why there was such a great difference between the grants given to the large general hospitals by the Sunday Fund and those given by the Saturday Fund. One of the systems of distribution must be wrong.

Other speakers also took part in the discussion.

The Chairman, in closing the discussion, explained that although the Sunday Fund had no committee of visitors, yet there were representatives of nearly every hospital on the Council of the Fund, and, therefore, the Fund had direct information as to the wants and necessities of each hospital.

Mr. Acland, in replying to the questions asked, said that the Rev. N. Bromley's objection seemed to be based upon the fact that the Saturday Fund made a certain deduction before commencing to distribute the money collected amongst the hospitals. He (the speaker) would like to know why not? Because a body of men had organised themselves for certain reasons, one of which was contributing to hospitals, surely no fraud was committed upon anybody if they deducted the amount necessary to carry out their other work before dividing up amongst the hospitals! As to Mr. Thies' suggestion, he was sure the committee of the Hospital Saturday Fund would welcome any assistance given them by the secretaries of hospitals. He agreed with Mr. Ryan that the economical management of a hospital could not be judged by taking the cost per bed and per patient, but would Mr. Ryan suggest a better system? As to whether the working man's guinea was of the same value as the rich man's, that was a question which it was difficult to discuss. Personally he believed it was, and he was quite satisfied it was so far as the Saturday Fund was concerned. That Fund issued from 20,000 to 22,000 letters every year, of which he supposed 90 per cent. reached the hospitals, and in return the Fund distributed a sum of money which worked out at about 15s. for each letter. No one, he was sure, would affirm that, taking all the patients together, in and out, casualties, lying-in cases, and cases visited at home, each patient cost the hospitals anything like 15s. If it did not the Saturday Fund as a whole did not impose on the hospitals as a whole. He thought he could best deal with the Earl of Stamford's question by giving the case of the hospital in which they stood. Last year that hospital treated 21,500 out-patients, including 10,000 casualties, and on the Fund's system they would receive one mark for every 2,000 casualties or out-patients, or altogether 10 marks for out-patients, which would work out at about £20. With regard to the marks given for the percentage of management and maintenance, to which Sir Henry Burdett alluded, the probability was that a case like that which he had mentioned would be excluded altogether under one of their other rules. As to the purchase of letters, he could not see where the iniquity came in. If all funds were blameless in this matter except the Saturday Fund, it might perhaps be fair to cast a stone at the Saturday Fund, but he had not observed that any gentleman who had spoken there that evening had referred to the rule of the Sunday Fund, which asks for letters on behalf of that Fund to the amount of half the number allowed to ordinary subscribers. Why was the Saturday Fund blamed for taking letters when it was done on a far larger scale by the Sunday Fund? (No, no!)

Sir Sydney H. Waterlow also replied. He quite agreed with those who felt that it would be difficult to amalgamate the two funds, but he failed to see why some common principle should not be arrived at by a conference as to the method of distribution. As to the question of subscribers'

letters, he still hoped to see the day when letters would be dispensed with. The careless way in which letters were given was what led to the most serious abuses of the out-patient department. He would gladly arrange a conference between the two funds on the letters question.

Votes of Thanks.

The Chairman moved a vote of thanks to Sir Sydney Waterlow and Mr. Acland.

Surgeon-Lieut.-Colonel J. Ince seconded, and the vote was unanimously accorded.

Votes of thanks to the chairman and to Charing Cross Hospital for the use of the board room were also given, and The meeting terminated.

